# P19-derived neuronal cells express H_1_, H_2_, and H_3_ histamine receptors: a biopharmaceutical approach to evaluate antihistamine agents

**DOI:** 10.1007/s00726-023-03273-6

**Published:** 2023-05-12

**Authors:** Mariela Gomez Perez, Georgiana Tanasie, Armelle Tchoumi Neree, Narjara Gonzalez Suarez, Clara Lafortune, Joanne Paquin, Lucia Marcocci, Paola Pietrangeli, Borhane Annabi, Mircea Alexandru Mateescu

**Affiliations:** 1 Department of Chemistry and CERMO FC Center, Université du Québec à Montréal, C. P. 8888, Montréal, QC H3C 3P8 Canada; 2 Department of Biochemical Sciences “A. Rossi Fanelli”, Sapienza University of Rome, 00185 Rome, Italy; 3 Chaire en Prévention et Traitement du Cancer, Université du Québec à Montréal, C. P. 8888, Montréal, QC H3C 3P8 Canada

**Keywords:** Antihistamine drugs, Biogenic amine, Catalase, Cimetidine, Ciproxifan, Copper-containing diamine oxidase, Desloratadine, Histamine, Histamine receptors, P19 embryonic carcinoma cells

## Abstract

Histamine is a biogenic amine implicated in various biological and pathological processes. Convenient cellular models are needed to screen and develop new antihistamine agents. This report aimed to characterize the response of neurons differentiated from mouse P19 embryonal carcinoma cells to histamine treatment, and to investigate the modulation of this response by antihistamine drugs, vegetal diamine oxidase, and catalase. The exposure of P19 neurons to histamine reduced cell viability to 65% maximally. This effect involves specific histamine receptors, since it was prevented by treatment with desloratadine and cimetidine, respectively, H_1_ and H_2_ antagonists, but not by the H_3_ antagonist ciproxifan. RT-PCR analysis showed that P19 neurons express H_1_ and H_2_ receptors, and the H_3_ receptor, although it seemed not involved in the histamine effect on these cells. The H_4_ receptor was not expressed. H_1_ and H_2_ antagonists as well as vegetal diamine oxidase diminished the intracellular Ca^2+^ mobilization triggered by histamine. The treatment with vegetal diamine oxidase or catalase protected against mortality and a significant reduction of H_2_O_2_ level, generated from the cells under the histamine action, was found upon treatments with desloratadine, cimetidine, vegetal diamine oxidase, or catalase. Overall, the results indicate the expression of functional histamine receptors and open the possibility of using P19 neurons as model system to study the roles of histamine and related drugs in neuronal pathogenesis. This model is less expensive to operate and can be easily implemented by current laboratories of analysis and by Contract Research Organizations.

## Introduction

Histamine is involved in various physiological and pathological processes. This biogenic amine stimulates gastric secretion and modulates the immune system activating inflammatory processes (Rocha et al. 2014; Branco et al. 2018). The excess of exogenous histamine can generate pseudo-allergies, whereas endogenous excess is associated with allergic phenomena and anaphylaxis (Kemp and Lockey 2002; Maintz and Novak 2007). Histamine modulates heart rate and contractility, vasodilatation, and paracellular permeability (Obara et al. 2020). It acts on neurons of the enteric nervous system (ENS) with effects on smooth muscle and intestinal contraction (Spencer and Hu 2020), and as a neuromodulator/neurotransmitter in the central nervous system (CNS). Histamine is involved in the sleep waking cycles, synaptic plasticity, learning, and in neurological disorders, such as migraine, neuroinflammation, epilepsy, and brain infarction (Haas et al. 2008; Yuan and Silberstein 2018), indicating the importance of drug development targeting the brain histamine system.

In the CNS, histamine synthesized by the enzymatic decarboxylation of L-histidine is stored in synaptic vesicles via vesicular monoamine transporter 2 in histaminergic neurons. Upon stimulation, histamine is released to extra neuronal spaces and exerts its effects through interactions with histamine receptors (H-receptors) (Brown et al. 2001; Duan and Wang 2010; Huang et al. 2018). Four histamine receptors (H_1_–H_4_) have been identified. In general, H_1_, H_2_, and H_3_ are distributed in various tissues, including the brain and the ENS, while H_4_ is found mostly on immune and intestinal cells. In the CNS, H_1_, and H_2_ are postsynaptic receptors present on neurons. Their activation by micromolar histamine levels excites neurons or potentiates excitatory inputs (Panula. 2021). Effects driven by histamine may interfere with cellular survival. Activation of H_1_ and H_2_ receptors in CNS neurons causes the release of calcium ions (Ca^2+^) from the endoplasmic reticulum followed by the production of arachidonic acid, nitric oxide (NO), and reactive oxygen species (ROS) (Molina-Hernández and Velasco 2008; Shahid et al. 2009; Panula et al. 2015). The production of ROS is controlled by defense systems which can involve superoxide dismutase, catalase (CAT), glutathione peroxidase, and glutathione reductase (Halliwell. 2007; Flora. 2009). H_3_ is a pre- and postsynaptic receptor mainly present on astrocytes and histaminergic neurons (Tiligada et al. 2011; Panula et al. 2015; Panula. 2021). Its activation by nanomolar histamine regulates the release and synthesis of this biogenic amine (Schwartz et al. 1991; Leurs et al. 2012). In these cells, histamine is internalized via organic cation transporter 3 (OCT3) and via plasma membrane monoamine transporters (PMAT) and finally metabolised by histamine N-methyltransferase (Duan and Wang 2010; Yoshikawa et al. 2019).

Antihistamine agents targeting H-receptors, such as desloratadine (DES), cimetidine (CIM), or ciproxifan (CPX), have been developed to alleviate histamine-induced dysfunctions (Leurs et al. 2005; Canonica and Blaiss 2011; Jafarzadeh et al. 2019). The spectrum of histamine functions in the CNS has been greatly expanded during the last decades. However, much remains to be learned about H-receptors with respect to the development of new and improved antihistamine agents for histamine dysfunctions that cause pathophysiological processes in neurons. The use of convenient cellular models in preclinical studies is suitable for the development of new drugs. P19 embryonal carcinoma cells have been used extensively to generate the P19 neuronal model due to the ability of these cells to differentiate into neurons in culture (McBurney et al. 1988). These P19 neurons can mimic those of the CNS based on the spectra of neurotransmitters and neuropeptides they produce (Staines et al. 1994; Ulrich and Majumder 2006). Previous works mentioned functional H_1_ receptors on undifferentiated P19 embryonal carcinoma cells (Bloemers et al. 1993), whereas our report is on P19 differentiated neurons. More recently, Molina-Hernández and Velasco (2008) have used E14 embryos extracted from pregnant Wistar rats to obtain multipotent neural stem cells. This is a longer and probably more expensive method. The advantage of cultured P19 differentiated neurons for serial drug evaluation of antihistamine drugs is that the whole procedure is shorter, easier to operate and avoids regulatory limitations for the use and handling of animals. Since the ENS contains a diversity of neurons, it is not excluded that P19 neurons could further serve as a model for the investigations of this system embedded in the lining of the gastrointestinal tract, which contains many of the neurotransmitters and neuropeptides found in the CNS (Furness. 2000; Mittal et al. 2017; Kulkarni et al. 2018; Spencer and Hu 2020).

Considering that neurons in the CNS and the ENS express H_1_, H_2_, and H_3_ receptors (Breunig et al. 2007), it was of interest to investigate to which extent P19 neurons can serve as a model to evaluate the effect of histamine and of antihistamine agents involved in the treatment of histamine-induced dysfunctions. Also, as histamine antagonists may have undesirable side effects, vegetal diamine oxidase (vDAO), such as that from *Fabaceae*, was suggested as an alternative treatment to alleviate various histamine-related dysfunctions associated with allergic conditions (Mondovi et al. 2013) and intestinal disorders (Neree et al. 2020). This enzyme (EC 1.4.3.22), in fact, can catalyze the oxidative deamination of histamine to the corresponding aldehyde, with the production of H_2_O_2_ and NH_3_. It also reduces neutrophil activation (Pietrangeli et al. 2020) and the consequent cellular oxidative damage (Masini et al. 2007). The aim of this work was thus to characterize the response to histamine of cultured P19 neurons and the modulation of this response by three common antihistamine agents (DES, CIM, and CPX) as well as by vDAO or CAT.

## Materials and methods

### vDAO purification and characterization

The vDAO was purified from *Lathyrus sativus* seedlings as previously reported (Blemur et al. 2016), but samples were homogenized in 50 mM phosphate buffer (pH 5.5) containing 200 mM NaCl. The vDAO samples were lyophilized and stored at −80° C until characterization. They contained 0.35 ± 0.05 mg protein/mg solid, as determined by the Bio-Rad assay with bovine serum albumin (BSA) as the standard. The vDAO-specific activity of 19 0.0 ± 2.6 U/mg solid was measured as reported (Jumarie et al. 2017). One enzyme unit was the amount of DAO enzyme able to catalyze the oxidation of 1 µmol of putrescine substrate per minute.

### Cell culture

Murine P19 embryonal carcinoma cells (1 × 10^6^) were cultured in 100 mM tissue-grade Petri dishes (Fisher Scientific, Montreal, QC, Canada) containing 10 mL complete alpha-Modified Eagle's Essential Medium (α-MEM; Invitrogen, Burlington, ON, Canada) supplemented with 10% (v/v) fetal bovine serum (FBS; Wisent Inc., St-Bruno, QC, Canada), 50 U/mL penicillin, and 50 µg/mL streptomycin (Sigma-Aldrich, Oakville, ON, Canada) at 37° C in a 5% CO_2_-95% humidified air atmosphere. Cells were passaged every 2 days by trypsinization (0.025% v/v trypsin-1 mM EDTA; Sigma-Aldrich, Oakville, ON, Canada) (Ducharme et al. 2010). The P19 cell line was obtained from Dr. M. W. McBurney (Université d'Ottawa, Ottawa, ON, Canada).

Neuronal differentiation was carried out by culturing Pl9 cells for 4 days in the presence of retinoic acid (RA) (McBurney et al. 1988). Briefly, 1.5 × 10^6^ cells were seeded in a 100 mm diameter bacteriological Petri dish (Fisher Scientific Montreal, QC, Canada) containing 10 mL of differentiation medium [DM: α-MEM supplemented with 5% (v/v) FBS, 5% (v/v) newborn calf serum (Wisent Inc., St-Bruno, QC, Canada), 50 U/mL penicillin, 50 µg/mL of streptomycin, and 0.5 μM all-trans-RA (Sigma-Aldrich, Oakville, ON, Canada)]. After 2 days, the differentiation medium containing RA was refreshed, and the cells were incubated for 2 more days. During the 4 days (D0–D4) of exposure to RA, the cells differentiated while forming floating spheroids in the DM. At D4, the cellular suspensions were collected and incubated at room temperature for about 5 min to allow the sedimentation of the spheroids which were then resuspended in phosphate buffer saline [PBS: NaCl 0.8% (w/v), KCl 0.02% (w/v), KH_2_PO_4_ 0.02% (w/v), Na_2_HPO_4_ 0.12% (w/v), and pH 7.3)] and sedimented again. After repeating this procedure for three times, the sedimented spheroids were dissociated by adding 2 mL of 0.025% (v/v) trypsin–1 mM EDTA. Then, 2 mL DM not containing RA was added to stop the reaction. Cell suspension was centrifuged for 1 min at 1000 g and then resuspended in synthetic medium [α-MEM supplemented with 5 µg/mL transferrin, 1 µg/mL insulin (Fisher Scientific Montreal, QC, Canada), 50 U/mL penicillin, and 50 µ/mL streptomycin].

### Cell treatment with antihistamine drugs, vDAO and CAT and H_2_O_2_

D4 resuspended cells were seeded (3.8 × 10^5^ cells/well) in gelatinized 12-well plates containing 2 mL of synthetic medium and incubated at 37° C in a 5% CO_2_ and 95% humidified air atmosphere for 2 days (D6). After this period, the culture medium was then renewed, and histamine, DES, CIM, CPX, vDAO, bovine liver catalase (CAT), putrescine (PUT), semicarbazide (SC), or hydrogen peroxide (H_2_O_2_) prepared in PBS, were added alone or in association with HA for the times and the final concentrations indicated in the figures. As controls, vDAO and CAT inactivated by heating at 60° C for 10 min (Andrews and Martin 1979; Mondovi et al. 1992) were also used. All agents, except vDAO, were purchased from Sigma-Aldrich (Oakville, ON, Canada). Cell phenotypic analysis, cell viability, and RT-PCR assay of H-receptors were then performed as indicated below.

### Cell phenotypic analysis by immunoblotting

For analysis of phenotypic markers, cells differentiated by RA for 4 days (D4) and collected as above reported were seeded in 4 mL of synthetic medium in 60 mm-diameter tissue-grade Petri at densities varying from 1.47 × 10^6^ to 0.37 × 10^6^ cells/Petri according to the duration of the subsequent cultures (D6–D12). Densities were adjusted to obtain similar final cell confluences between cultures while avoiding reseeding which could have disturbed cell culture composition. The synthetic medium was refreshed every 2 days. At different intervals of time, cells were washed in PBS and lysed for 30 min, 4° C, in radioimmunoprecipitation buffer [150 mM NaCl, 50 mM Tris, pH 7.6, 1% (w/v) Nonidet P-40, 0.5% (w/v) deoxycholate, and 0,1% (w/v) sodium dodecylsulfate (SDS) (w/v)] containing 1 mM sodium orthovanadate, 1 mM NaF, and 1/300 protease inhibitors Cocktail-1 (Sigma-Aldrich, Oakville, ON, Canada). The resulting cell protein extracts were analyzed for their protein contents using a bicinchoninic acid microassay kit (Pierce Chemical Co., Rockford, IL, USA), and submitted to SDS-polyacrylamide gel electrophoresis, electron transfer, and immunoblotting procedures as described by Ducharme et al. (2010) with a few modifications. Briefly, protein samples migrated for 15 min at 100 V and 1.25 h at 150 V through 10% polyacrylamide gels and were then transferred from gels onto polyvinylidene difluoride membranes for 35 min at 15 V in transfer buffer [10 mM Tris, 96 mM glycine, 0.01% SDS (w/v), and 20% (v/v) methanol]. After transfer, membranes were washed and incubated with antibodies in TBS-Tw buffer [150 mM NaCl, 50 mM Tris–HCl, pH 7.6, and 0.1% Tween-20 (v/v)] supplemented with 3% (w/v) bovine serum albumin. Primary antibodies were used in the indicated dilutions: mouse IgG_1_ anti-neurofilament-M (NF-M; 1/1000; Cat. No. 2838; Cell Signaling Technology, Danvers, MA, USA); mouse IgG anti-glial fibrillary acidic protein (GFAP; 1/400; Cat. No. G3893; Sigma-Aldrich, Oakville, ON, Canada); rabbit IgG anti-synaptophysin (SYP; 1/200; Cat. No. sc9116; Santa Cruz Biotechnology, Santa Cruz, CA, USA), and mouse IgG anti-β-Actin (1/10000; Cat. No. A5441; Sigma-Aldrich, Oakville, ON, Canada). Secondary antibodies, conjugated to horseradish peroxidase were from Jackson ImmunoResearch Laboratories, Mississauga, ON, Canada (Cat. Nos. 115–035-062 and 711–035-152; 1/2500 each). Immune complexes were revealed with Immobilon Western Chemiluminescent Substrate (Millipore, Nepean, ON, Canada) and exposed to HyBlot CL films (Denville Scientific Inc., Toronto, ON, Canada) or to a Fusion FX7 imaging system (MBI, Montreal, QC, Canada).

### Cell viability assay

The viability of the cells following treatments was measured by the neutral red (NR) assay based on the accumulation of the dye via active transport in the lysosomes of live cells (Perez et al. 2017). At the end of the treatments, above indicated, cells were delicately pre-washed with PBS, and then, 1 mL of 20 mM HEPES, pH 7.4 containing 138 µM NR, 140 mM NaCl, 4 mM KCl, 1.8 mM CaCl_2_, 0.8 mM MgCl_2_, and 20 mM D-glucose was added to each culture well. After 2 h of incubation at 37° C and under 5% CO_2_, the solution was removed, and cells were washed rapidly with 1 mL of solution containing 1% formaldehyde (v/v) and l% CaCl_2_ (w/v). NR contained in the cells was then extracted by incubation for 10 min, under agitation, with 1 mL of ethanol:H_2_O:acetic acid solution (50:49:1), and the absorbance read at 540 nm using a microplate reader (Molecular SpectraMax EM Microplate Reader, CA, USA). The viability of cells was calculated as percentages, referring to the absorbance of untreated living cells. All reagents were purchased form Sigma-Aldrich (Oakville, ON, Canada). The half maximal effective concentration (EC50) was estimated as the concentration of HA which induced a response halfway between maximum of viability and the baseline when viability remained constant after a 48 h of exposure to HA.

### RT-PCR assay of H-receptors

At the end of exposure time to histamine alone or in association with DES, CIM, or CPX, the total RNA was isolated from the cell pellets using 1 mL TRizol reagent per well (LiTechnologiesies, Gaithersburg, MD, USA). Next, 1–2 μg of RNA was reverse-transcribed to cDNA using the high-capacity cDNA reverse transcription kit (Applied Biosystems, Foster City, CA, USA) and following the manufacturer’s instructions. Real-time polymerase chain reaction (RT-PCR) was then performed to determine the gene expression level of the H_1_, H_2_, and H_3_ receptors using the SsoFast EvaGreen Supermix (Bio-Rad, Hercules, CA, USA), and 30 cycles of amplification in a CFX Connect Real-Time System (Bio-Rad, version 2.1). The resulting amplification products were detected by measuring the fluorescence elicited by the binding of the SYBR Green dye to the double-stranded DNA. QuantiTect primers for H_1_ (Hrh1_PPM04806B), H_2_ (Hrh2_PPM04805A), H_3_ (Hrh3_QT00158375), and H_4_ (Hrh4_PPM04894A) receptors were purchased from Qiagen (Montreal, QC, Canada). The RPS18 (RRN18S_QT00199367) and peptidylprolyl isomerase A (PPIA_QT01866137) were included as house-keeping genes. Finally, RT-PCR amplicons were electrophoresed, adding molecular weight standards (FroggaBio, Concord, ON, Canada) on a 2% agarose gel, visualized with GreenGlow™ (Denville Scientific Inc., Saint-Laurent, QC, Canada) and using a ChemiDoc MP Imaging System (Bio-Rad, Hercules, CA, USA).

### Cellular Ca^2+^ mobilization

Cells, grown in synthetic medium for 2 days (D6), were exposed for 1 h to Hank’s Balanced Salt Solution containing 10 mM HEPES, pH 7.2 (HBBS-HEPES) and 1 µM Fluo-4 acetoxymethyl ester calcium probe (Fluo-4 AM; Thermo Fisher Scientific, Montreal, QC, Canada), 1 µM pluronic acid F127 (Thermo Fisher Scientific, Montreal, QC, Canada), and 2.5 mM probenecid (Sigma-Aldrich, Oakville, ON, Canada-Aldrich) at 37° C in a 5% CO_2_-95% humidified air atmosphere. Then, the cells were washed three times with HBSS-HEPES and maintained in 1 mL of the same buffer for 30 min. Cells were then added with 100 µM histamine in the absence or presence of 5 µM DES, 0.1 µM CIM, or 0.4 µM vDAO, and fluorescent images were acquired by a confocal microscopy by measuring the fluorescence of Fluo4-Ca^2+^ signal at 525 nm, upon excitation at 488 nm. Nikon A1 confocal microscope equipped with a 10X objective (Nikon Canada, Mississauga, ON, Canada) was used. The acquisition was operated in kinetic scan mode, at 2.5 min with a rate of 2 s per scan, a pinhole diameter of 11.9 µm, a detector sensibility or high voltage of 45, and an offset of -5. The ImageJ software (Wayne Rasband National Institutes of Health, USA) was used to quantify the Ca^2+^-fluorescence. Results are expressed as area under the curve (AUC) for the first 120 s following the addition of HA.

### H_2_O_2_ spectrofluorimetric assay

The generation of H_2_O_2_ was measured using a spectrofluorimetric assay based on the oxidation of homovanillic acid (HVA) under the catalysis of horseradish peroxidase (HRP) forming a fluorescent dimer in the presence of H_2_O_2_ (Baggiolini et al. 1986). Briefly, Day 6 P19 neuron cultures, obtained as described above, were delicately pre-washed with PBS and received 1.75 mL of a reaction mixture containing HVA (100 µM)-HRP (1U/mL) in HBSS. The reaction was started by the addition of 0.25 mL of HA (100 µM in HBSS) alone or in association with DES (5 µM), CIM (1 µM) or CPX (0.01 µM). The blank control cultures received HBSS alone and the positive control cultures received HBSS containing 10 nmol H_2_O_2_. Cultures treated with DES (5 µM), CIM (1 µM), or CPX (0.01 µM) in the absence of HA were also included for comparison. After a 4 h of incubation at 37 °C, 5% CO_2_, the reaction was stopped with 0.25 mL of 0.1 M NaOH (pH 12). The reaction mixtures were collected and centrifuged for 10 min at 1200 g, and their fluorescence measured at 25 °C in a PerkinElmer LS45 fluorimeter (PerkinElmer, Waltham, MA, USA), using 315 nm (excitation) and 425 nm (emission) wavelengths. The standard curves were performed in the absence of cells. The standard solutions were prepared by the addition of 0.25 mL HBSS alone (blank) or containing 0.1 to 10 nmol H_2_O_2_, to 1.75 mL of HBSS containing HVA (100 µM)-HRP (1U/mL). The reaction was stopped after 4 h by the addition of 0.25 mL of 0.1 M NaOH. The fluorescence value of each culture sample and standard solution was corrected from that of its own blank before its report to the standard curve. Upon verification, there was no background fluorescence in these additional blanks: H_2_O_2_ ± cells, HVA ± cells, HRP ± cells, (HVA + HRP) ± cells, (H_2_O_2_ + HVA) ± cells, and (H_2_O_2_ + HRP) ± cells.

### Statistics

All experiments used a minimum of three replicates. Where relevant, data are expressed as the mean ± SD. Statistical tests were performed with the GraphPad software, using either one-way, two-way ANOVA (for comparison between three groups) or two-tailed Student’s t test (for comparison between two groups). Differences were deemed statistically significant when the associated P value was lower than 0.05 (P < 0.05).

## Results and discussion

### Differentiation of cultured P19 cells into neurons by retinoic acid

**Fig. 1:**
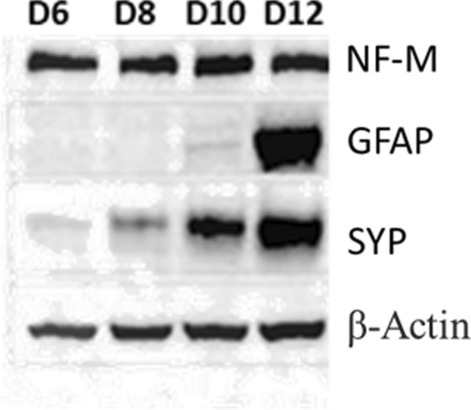
Time-course of the expression of phenotypic markers in P19 neuron cultures. A representative immunoblot of three independent studies. β-Actin was used as a protein loading control

The treatment of mouse P19 embryonal carcinoma cells with RA for 4 days have been reported to induce their differentiation into a population of cells consisting of neurons and other cell types, such as fibroblasts and glial cells. Neurons express a variety of neuronal markers, develop neurite processes presenting characteristics of axons and dendrites, and form synapses (Staines et al. 1994; Poirier et al. 2006). Immunoblotting analysis of NF-M neuronal marker showed the presence of neurons from D6 to D12, the last day analyzed (Fig. 1).

Neurons mature in these cultures as indicated by the increasing levels of SYP synaptic marker (Fig. 1). The GFAP glial marker was almost undetectable until D10 indicating a very low number, if not the absence of astrocytes in the cell populations during this time interval (Fig. 1) This is in line with our reported immunofluorescence studies, showing that D10 RA-treated cultures contain mainly neurons, since about 95% of the cells expressed the neuronal nuclear marker NeuN and about 5% were GFAP positive (Poirier et al. 2006). As our objective was to characterize the response to histamine of cultured P19 neurons in absence of astrocytes, the following studies were carried out with D6 cultures. This is pertinent to the fact that astrocytes participate in the regulation of histamine metabolism and function (Jurič et al. 2016; Otsuka and Naganuma 2022).

### Loss of cell viability in P19 neurons treated with histamine

**Fig. 2:**
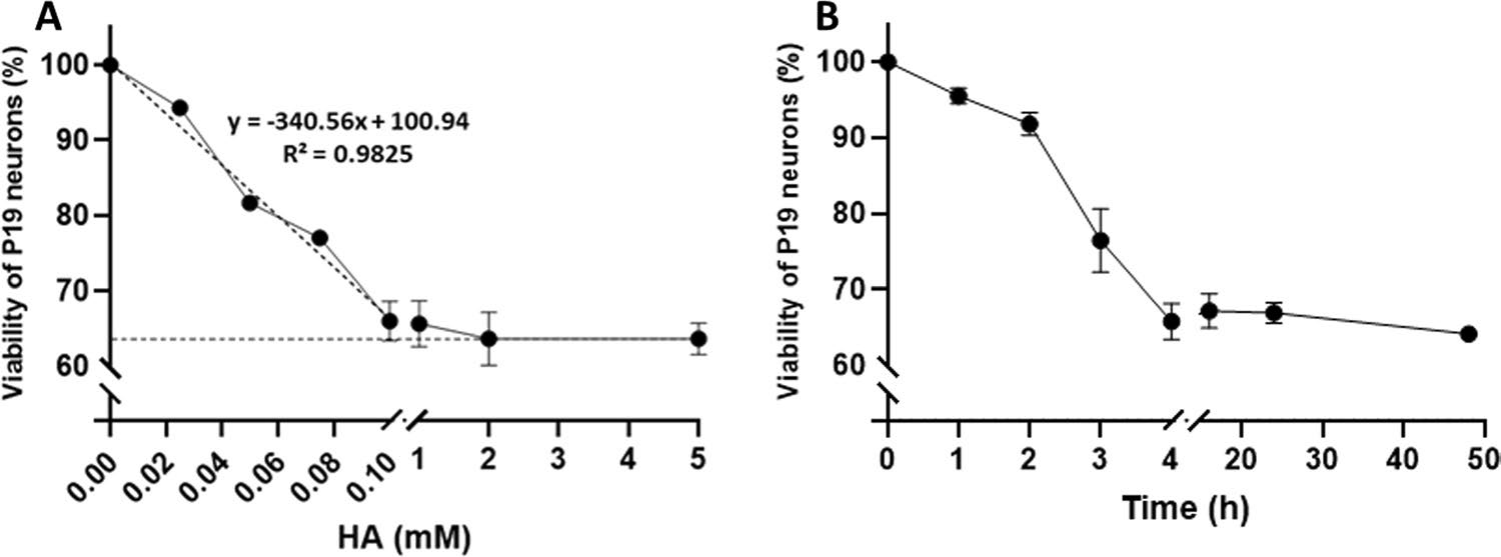
Effect of histamine on P19 neurons viability: **A** D6 P19 neurons were treated for 48 h with various concentrations of histamine (HA) and analyzed for viability. The dotted lines show the linear relationship of histamine action at concentrations ≤ 100 µM, and the plateau effect at higher concentrations. **B** Time-course of P19 neurons viability upon exposure to 100 µM histamine at D6. Data are means ± SD of three independent experiments and reported as percentages of untreated control cultures

The incubation of D6 P19 neurons with histamine for 48 h caused a loss of viability (Fig. 2A). Indeed, viability dropped to 65% in a histamine-dependent concentration for up to 100 µM histamine. The EC50 value of this histamine effect was 54.1 ± 1.2 µM, that is the concentration for the median value between 100 and 65% viability (or between 0 and 35% mortality). This calculation considers that the loss of viability remained constant (64 ± 2%) for concentrations equal to or higher than 100 µM histamine. This behavior differs from what was previously observed with Caco-2 cells, in which the loss of viability consistently increased with the concentration (Jumarie et al. 2017).

The results here reported suggest that 65% of the cells remained unaffected or cannot respond to histamine action. This would be in line with the fact that, like primary neuronal cultures, P19 neuronal cultures contain mixed populations of neurons (McBurney et al. 1988). Another possibility is that the H-receptors are progressively impacted by the amount of histamine administered. When the whole amount of H-receptor is saturated, a larger amount of histamine would not generate a further decrease of viability. Accordingly, in the present work, 100 µM histamine was chosen as the reference concentration to carry out subsequent tests.

The time-dependent effect of 100 µM histamine was also analyzed. As reported in Fig. 2B, histamine caused a marked linear decrease in cell viability during the first 4 h of exposure. Then, cell viability remained constant (about 65%) for up to 48 h. It is not excluded that the first step of HA impacting the H-receptors was followed by a period related to an arrest of signalization. The effect of histamine on neuronal viability could involve its metabolites or its receptors. This possibility has been next evaluated.

### No involvement of DAO activity in the histamine-induced loss of cell viability

**Fig. 3:**
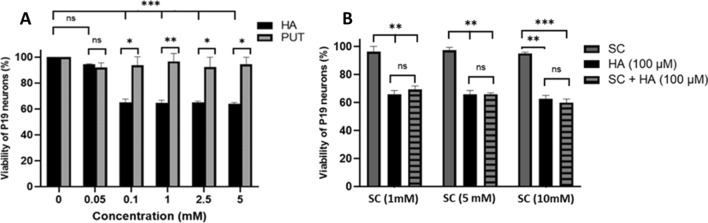
Effect of putrescine and semicarbazide in relation with the action of histamine on P19 neurons viability. Cells were incubated for 4 h in the presence of histamine (HA) or putrescine (PUT) at different concentrations (**A**), or in the presence of 100 µM of histamine with or without semicarbazide (SC) at various concentrations (**B**). Data are means ± SD of three independent experiments in duplicate and reported as percentages of untreated control cultures (*n* = 3; *ns* non-significant; *P < 0.05, **P < 0.01, ***P < 0.001; two-way ANOVA multiple comparisons)

The histamine effect on the viability of P19 neurons could have been related to: (*i*) the oxidative deamination of this biogenic amine by an endogenous DAO which would have produced cytotoxic H_2_O_2_, imidazole-4-acetaldehyde and NH_3_ or (*ii*) the activation of H-receptors leading to the release of ROS, and nitric oxide (NO). To investigate the possible involvement of an endogenous DAO, P19 neurons were treated with putrescine (PUT) and semicarbazide (SC) (Fig. 3). The DAO can metabolize not only histamine but also putrescine (the substrate for which DAO displays the highest rate of enzymatic oxidation (Pietrangeli et al. 2007)). Also, as a copper enzyme, DAO is inhibited by semicarbazide (Obata. 2002).

Figure 3A presents the comparative effects on cell viability of HA and PUT used at the same concentrations. PUT did not affect the viability of cells despite its higher degradation by DAO. As already observed above, the viability of cells decreased to 65% for histamine concentrations ≥ 0.1 mM. On the contrary, PUT presented good biocompatibility, preserving cell viability even at concentrations up to 5 mM. These data suggest that the decreased viability was specifically related to histamine only and not to putrescine. Moreover, if an endogenous copper-containing DAO was present on P19 neurons, this DAO would have modulated the viability of P19 neurons, and this modulation would have been modified in the presence of SC known as a specific inhibitor of copper-containing enzymes and is largely used to confirm the presence of these enzymes in biological systems (Obata. 2002).

Practically, Fig. 3B shows that SC itself (1–10 mM) was not toxic to cells. Furthermore, SC did not prevent the loss of cell viability induced by histamine since the viability remained at about 65%. Therefore, the results with SC provided an argument supporting the absence of an endogenous DAO and agreed with other data showing that DAO expression in the CNS is low or absent (Yoshikawa et al. 2019). Data of this section indicated that the toxic effect of histamine was not related to its degradation by an endogenous DAO but involved other cellular mechanisms such as activation of receptors.

### Involvement of H-receptors in histamine-induced loss of cell viability

**Fig. 4:**
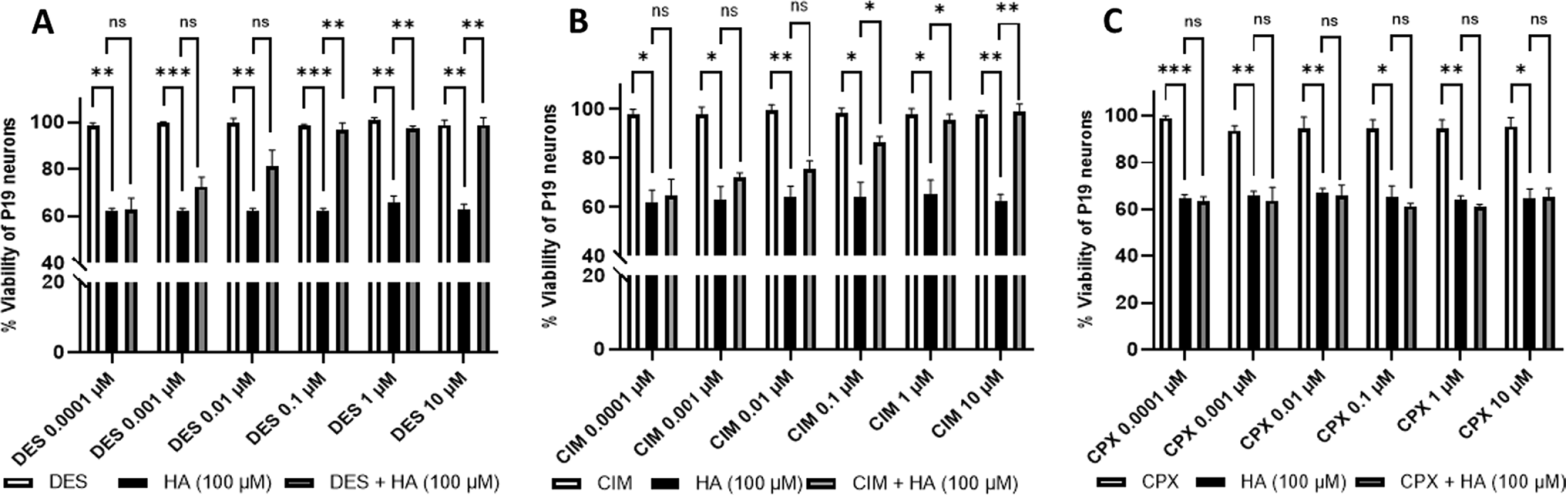
Effect of histamine (HA, 100 µM) on cell viability after 4 h of treatment in the absence or presence of the antihistamine agents: Desloratadine (DES) (**A**), Cimetidine (CIM) (**B**), and Ciproxifan (CPX) (**C**). Notice the absence of an effect on viability for DES, CIM, and CPX used alone (**A, B, C**). Data are means ± SD of three independent experiments in duplicate (*n* = 3; *P < 0.05, **P < 0.01, ***P < 0.001; two-way ANOVA multiple comparisons)

To evaluate the involvement of H-receptors in histamine-induced loss of cell viability, P19 neurons were treated with H_1_ (DES), H_2_ (CIM), or H_3_ (CPX) antagonists for 4 h, in the presence of 100 µM histamine (Fig. 4). DES is known as a non-competitive antihistamine agent acting on H_1_ by binding to a site different from that of histamine-binding site (Agrawal 2001) and CIM is a competitive inhibitor of H_2_ (Jafarzadeh et al. 2019). CPX is considered as a potent and selective H_3_ antagonist/inverse agonist that binds to the histamine-binding site on the receptor (Leurs et al. 2005). None of the three antihistamine agents exerted a toxic effect by themselves (Fig. 4).

Both DES and CIM decreased the histamine-induced loss of viability in an apparent concentration-dependent manner, with a complete abolition of the cytotoxic effect at concentrations greater than 0.1 µM (Fig. 4A, B). The apparent EC50 values for this protection were about 0.044 ± 0.01 µM and 0.059 ± 0.005 µM, respectively. Differently, CPX did not alleviate the loss of cell viability triggered by histamine (Fig. 4C). These results point to the activation of H_1_ and H_2_, but not H_3_, as being involved in the neuronal cytotoxic effect of histamine.

### Histamine-induced cellular Ca^2+^ mobilization

**Fig. 5:**
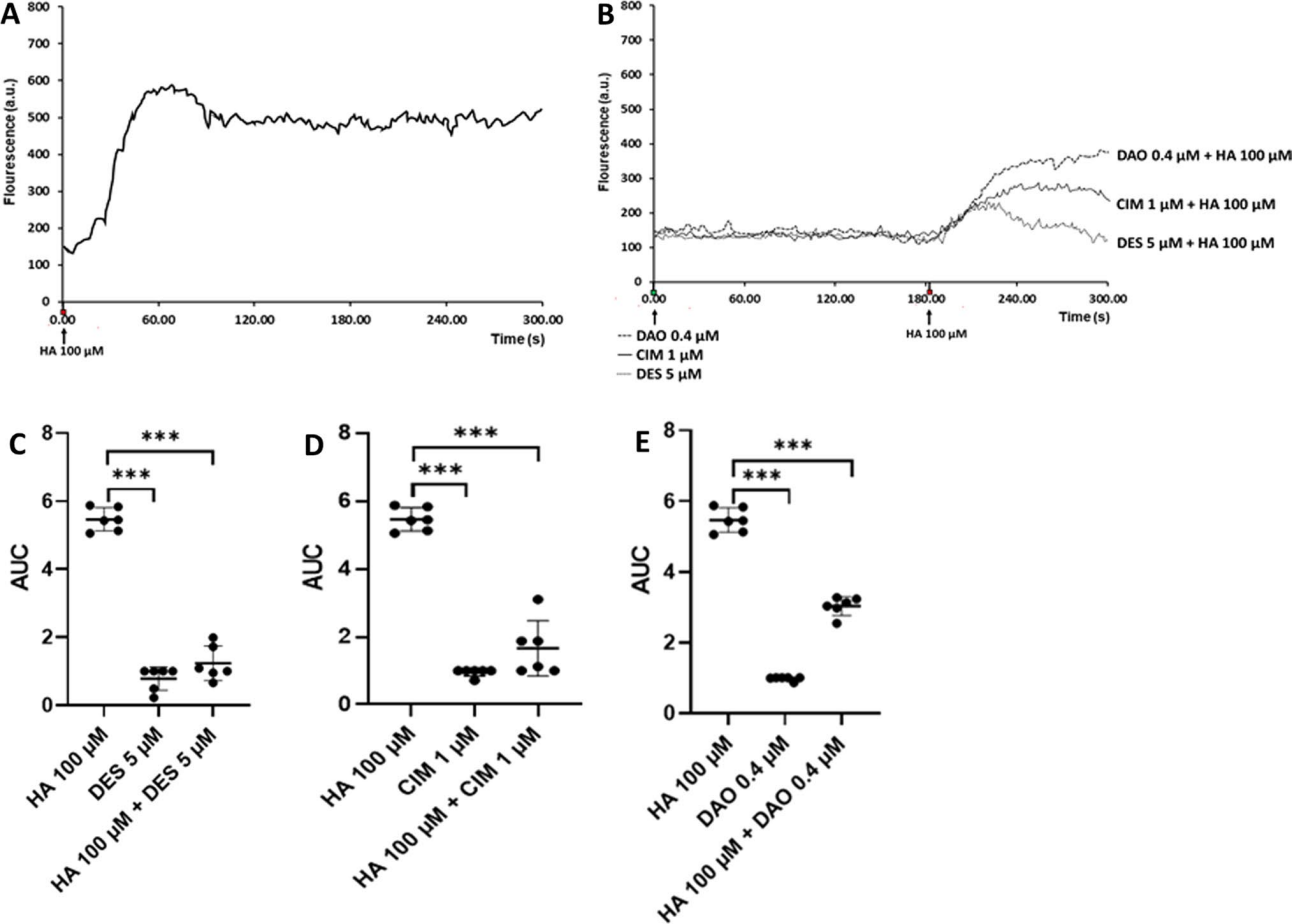
Effect of histamine, antihistamine drugs, and vDAO on intracellular calcium levels in P19 neurons. Neurons were pre-loaded with the Fluo-4AM calcium probe before treatment with the different agents. Time-course of fluorescence profile related to Ca^2+^ mobilization induced by histamine (HA, 100 µM) stimulation alone (**A**) or following a 180 s pretreatment with DES (5 µM), CIM (1 µM), or vDAO (0.4 µM) (**B**) as measured by real-time confocal microscopy. The quantified area under the curve (AUC) was obtained by ImageJ software (**C, D, E**). Data are expressed as means ± SD of at least three experimental runs in duplicate (*n* = 3; ***P < 0.001; two-way ANOVA multiple comparisons)

The activation of H_1_ and H_2_ has previously been reported to trigger Ca^2+^ mobilization from the endoplasmic reticulum stores (Jones and Kearns 2011) through the activation of the phospholipase-C pathway (Panula et al. 2015; Obara et al. 2020). The activation of Gα_i/o_ proteins may lead to the mobilization of intracellular Ca^2+^ levels upon the activation of H_3_ (Bongers et al. 2007). Since, the H_3_ receptor did not seem to be involved in the loss of neuron viability by histamine stimulus (Fig. 4C), the modulation of Ca^2+^ mobilization under histamine stimulation by CPX was not included. The concentrations used of DES and CIM were higher than their respective Kd values (Kd = 1.1 for DES (Anthes et al. 2002); Kd = 0.8 μM for CIM (Khateb et al. 1995), to afford a possible saturation of receptors by these antihistamine agents. The fluorescence measurements of intracellular Ca^2+^ level showed that Ca^2+^ mobilization was induced by exposure to histamine (Fig. 5A). This was significantly attenuated when cells were co-treated with histamine and the antihistamine DES (Fig. 5B, C) or CIM (Fig. 5B, D). These results support the hypothesis of the presence of H_1_ and H_2_ in P19 neurons and their involvement in histamine-induced Ca^2+^ mobilization. The Ca^2+^ signal induced by histamine was also lowered by the addition of vDAO (Fig. 5B, E) which should be due to the oxidative decomposition of histamine by this enzyme. Overall, it appears that P19 neurons express functional H_1_ and H_2_ receptors and their inhibition or blockage is sufficient to abolish the histamine-induced loss of neuronal viability.

### Expression of H_1_, H_2_, and H_3_ receptors in P19 neurons

**Fig. 6:**
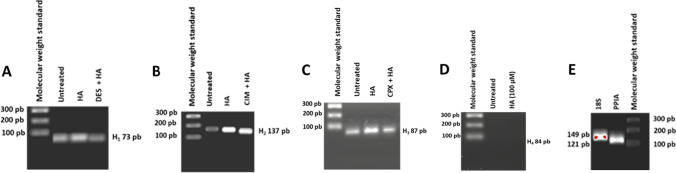
Agarose gels of H_1_ (**A**), H_2_ (**B**), H_3_ (**C**), and H_4_ (**D**) amplicons obtained by RT-PCR analysis. P19 neurons were incubated for 4 h with histamine (HA, 100 µM) in the absence or presence of DES (1 µM), CIM (0.1 µM), or CPX (0.01 µM). The 18S was used as endogenous RNA standard and peptidylprolyl isomerase A (PPIA) as an internal standard (**E**). The gels are representative of three repeated experiments

We used RT-PCR to address whether P19 neurons do express H-receptors (Fig. 6). The presence of H_1_, H_2_, and H_3_ was clearly revealed by the generation of specific amplicons of the expected sizes (73 bp for H_1_, 137 bp for H_2_, and 87 bp for H_3_). The expression of the H_1_ receptor has been reported in undifferentiated P19 cells (Bloemers et al. 1993), but, to the best of our knowledge, this is the first report of the expression of H_1_, H_2_, and H_3_ receptors in P19 neurons. On the contrary, H_4_ seemed to not be expressed in view of the absence of specific amplicons (84 bp for H_4_) (Fig. 6D).

### Modulation of histamine effect on P19 neurons by exogenous diamine oxidase and catalase

**Fig. 7:**
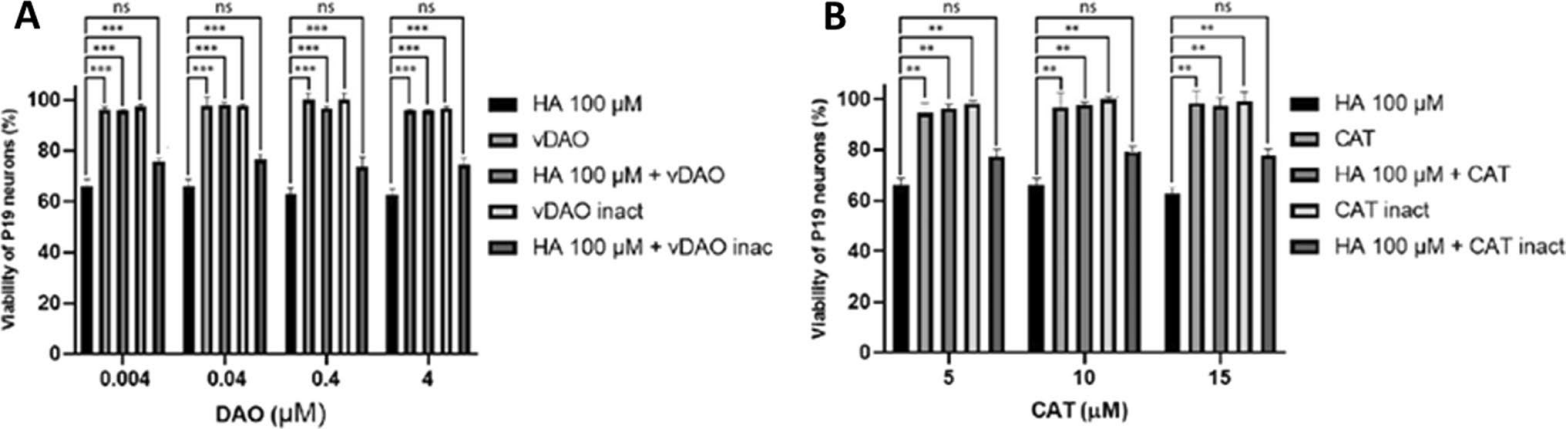
Effect of vDAO and CAT on histamine-induced loss of neuron viability. P19 neurons were treated for 4 h with 100 µM histamine (HA) in the absence or presence of active or heat-inactivated vDAO (**A**) or CAT (**B**), at various concentrations. The results (means ± SD of three independent experiments in duplicate) are reported as percentages of untreated cells (*n* = 3; **P < 0.01, ***P < 0.001; two-way ANOVA multiple comparisons

To evaluate whether the effect of histamine on cell viability is derived from the H_2_O_2_ produced from its own interaction with H-receptors, we next treated cultured P19 neurons with vDAO (acting on histamine) or with CAT (decomposing H_2_O_2_). Figure 7A shows that vDAO alone, whether active or inactivated, did not alter cell viability. When associated to histamine, vDAO under its active form (even at nanomolar concentrations), almost completely canceled the toxic effect of histamine. This protection tended to slightly diminish at higher concentrations, possibly due to the production of H_2_O_2_ by its oxidase reaction, and was abolished in the case of inactivated vDAO, indicating that the involvement of its enzymatic activity is requested for this protection.

Figure 7B shows that active and inactivated CAT alone did not affect cell viability and that active CAT protected P19 neurons against histamine toxicity. These results suggest an involvement of H_2_O_2_ generated by the cells in histamine toxicity. The activation of H-receptors triggers a series of reactions leading to the generation of ROS, NO, and H_2_O_2_ that are detrimental to cells, since they can cause in situ oxidative damage to cell structures or components, such as membranes, proteins, and DNA. Previous studies have shown that histamine induced the production of ROS in N9 microglial cell line and in primary murine microglia cells via the activation of H-receptors by 100 μM histamine (Rocha et al. 2016). This concentration was the same as that used in this report. Also, Medina et al. (2009) showed that histamine (100 μM) stimulated the production of H_2_O_2_ in WM35 melanoma cells and this effect was reversed by CAT treatment.

**Fig. 8:**
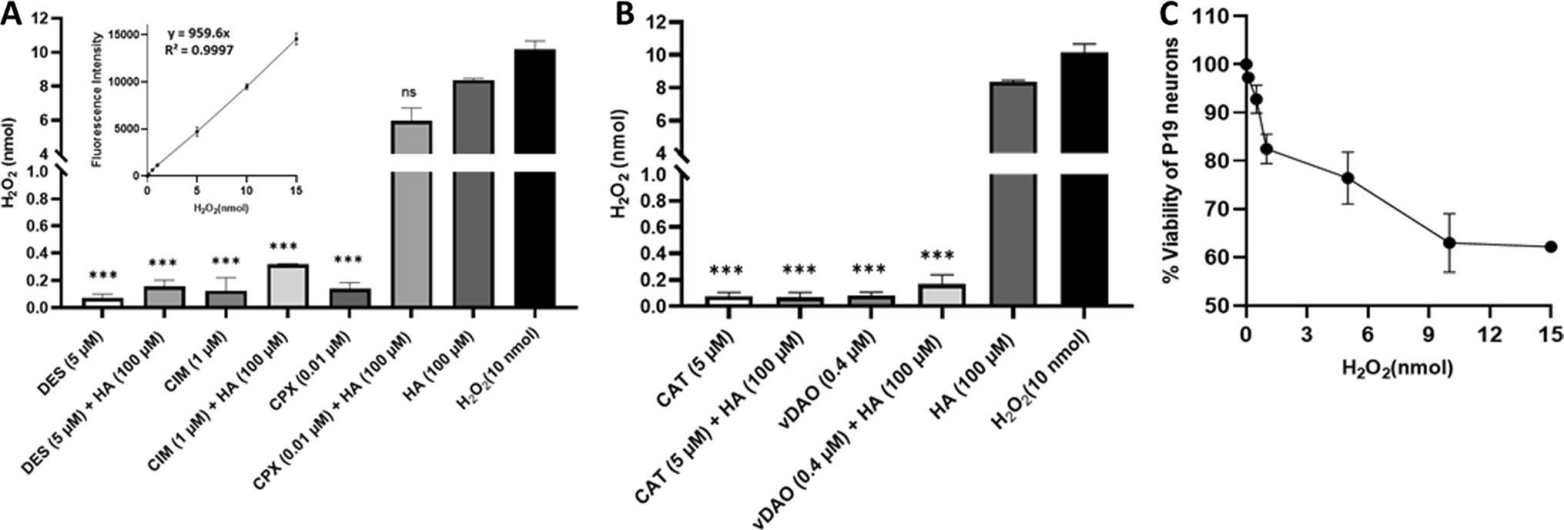
Production and toxic effect of the H_2_O_2_ in P19 neuronal cultures. Determination of H_2_O_2_ released in vitro by P19 neurons after 4 h of treatment with histamine (HA, 100 µM) in the presence or in the absence of the antihistamine agents: **A** Desloratadine (DES), Cimetidine (CIM), or Ciproxifan (CPX), or **B** Catalase (CAT) or vDAO, using a H_2_O_2_ standard curve (inset in A). The 10 nmol H_2_O_2_ standard were included as positive control. It was noticed the low levels of H_2_O_2_ production when DES, CIM, CPX, CAT, and vDAO were used alone. **C** Effect of H_2_O_2_ (0–15 nmol) on cell viability. The results are means ± SD of three independent experiments done in duplicate (*n* = 3; ***P < 0.001; one-way ANOVA multiple comparisons)

To investigate if histamine should stimulate the production of H_2_O_2_ in P19 neurons, these cells were treated with histamine (100 µM) alone or in association with antihistamine drugs, vDAO or CAT (Fig. 8). The fluorometric measure of oxidation product of HVA catalyzed by HRP was used to quantify the H_2_O_2_ produced by cells. The medium recuperated from cells treated with histamine (100 µM) contained 8.36 ± 0.08 nmol of H_2_O_2_ (Fig. 8A, B). An important reduction of the amount of H_2_O_2_ was observed when cells were exposed to histamine in association with DES or CIM at the concentrations showed in Fig. 8A. In contrast, CPX (H_3_ antagonist) was not able to reduce the generation of H_2_O_2_ (Fig. 8A). The inhibition or blockage of H_1_ and H_2_ receptors by DES or CIM, respectively, was also sufficient to avoid the loss of neuronal viability (Fig. 4A, B) as well as the reduction of Ca^2+^ signal, both induced by histamine (Fig. 5A–D).

The vDAO and CAT also decreased the production of H_2_O_2_ (Fig. 8B). CAT could protect cells against histamine-mediated loss of viability by scavenging H_2_O_2_, while exogenous vDAO, although a generator of H_2_O_2_, could provide protection by decreasing the amount of histamine capable of binding to H-receptors. In aerobic organisms, CAT, as an enzyme involved in the scavenging of ROS, transforms H_2_O_2_ into molecular oxygen and H_2_O. It is important to note that H_2_O_2_ is a stable nonradical oxidative species that can easily cross biological membranes and move from the interior to the exterior of the cell (Flora 2009) where it can be decomposed by CAT (Medina et al. 2009). The viability of P19 neurons is affected by H_2_O_2_ quantities as those produced by histamine treatment (Fig. 8C).

## Conclusions

The results provide evidence that P19 neurons express functional H_1_, H_2_, and H_3_ and that their exposure to histamine results in a loss of cell viability. The observed histamine-dependent cytotoxic effect was prevented by the treatment with DES and CIM (H_1_ and H_2_ antagonists) as well as by the addition of vDAO or CAT but not by H_3_ antagonist. The H_4_ seemed to not be expressed on these cells. The vDAO likely protects by decreasing extracellular histamine levels, and CAT by scavenging H_2_O_2_ escaped from the cells following activation of H-receptors. Also, DES and CIM can significantly reduce the amount of H_2_O_2_ released by cells treated with histamine. P19 neurons appear as sensitive to histamine (being affected by low concentrations of this compound), pointing to their usefulness as a pertinent model in the search for histamine roles in neuronal system. Furthermore, P19 neurons, easy to cultivate, can be used in the investigation of pharmaceuticals such as antihistamine agents, and neuropharmaceuticals, particularly for histamine-related neuronal dysfunctions. As a perspective, they can be considered in the biopharmaceuticals studies to evaluate novel antihistamine drugs or the association of antihistamine drugs with other bioactive agents in histamine neural neuronal pathogenesis. The major advantages of the proposed approach consist in the fact that various antihistamine drugs can be simply and directly evaluated in the presence of histamine. The robustness of P19 neurons together with their easy handling make the P19 neuronal model an interesting choice for rapid *in vitro* screening of antihistamine drugs. This model presents properties like those of cultured mammalian brain and it is less expensive to operate than primary cell cultures. This model can be easily implemented by current laboratories of analysis and by Contract Research Organizations (CROs).

## Abbreviations

AUC: Area under the curve
Ca^2+^: Calcium ions
CAT: Catalase
CIM: Cimetidine
CNS: Central nervous system
CPX: Ciproxifan
D: Day
DAO: Diamine oxidase
vDAO: Vegetal DAO
DES: Desloratadine
DM: Differentiation medium
ENS: Enteric nervous system
FBS: Fetal bovine serum
GFAP: Glial fibrillary acidic protein
H_1_: Histamine H_1_ receptor
H_2_: Histamine H_2_ receptor
H_3_: Histamine H_3_ receptor
H_4_: Histamine H_4_ receptor
H-receptor: Histamine receptors
HA: Histamine
HBSS: Hank’s balanced salt solution
H_2_O_2_: Hydrogen peroxide
HEPES: 4-(2-Hydroxyethyl)-1-piperazineethanesulfonic acid
HRP: Horseradish peroxidase
HVA: Homovanilic acid
NF-M: Neurofilament M
NO: Nitric oxide
NR: Neutral red
PBS: Phosphate-buffered saline
PUT: Putrescine
RA: Retinoic acid
ROS: Reactive oxygen species
RT-PCR: Real-time polymerase chain reaction
SC: Semicarbazide
SDS: Sodium dodecylsulfate
SYP: Synaptophysin
